# A case report of the rapid dissemination of Kaposi’s sarcoma in a patient with HIV

**DOI:** 10.4102/phcfm.v5i1.526

**Published:** 2013-06-25

**Authors:** Indiran Govender, Mogakgomo H. Motswaledi, Langalibalele H. Mabuza

**Affiliations:** 1 Department of Family Medicine & Primary Health Care, University of Limpopo, Medunsa Campus, South Africa; 2 Department of Dermatology, University of Limpopo, Medunsa Campus, South Africa

## Abstract

**Introduction:**

Kaposi’s sarcoma is the most common HIV-associated neoplastic disease. In most cases it starts on the skin and later spreads to other visceral organs. We reported a case of HIV-associated cutaneous Kaposi’s sarcoma which rapidly progressed to involve the visceral organs within a few weeks and resulted in fatality.

**Case presentation:**

A 21-year old man who recently started antiretroviral therapy developed disseminated Kaposi’s sarcoma with a right-sided pleural effusion. Chest x-ray confirmed the effusion which was tapped for diagnostic purposes. Biopsy confirmed Kaposi’s sarcoma. He insisted on being discharged so that he could visit a traditional healer.

**Management and outcome:**

Despite antiretroviral therapy and supportive management his condition deteriorated rapidly and he died within a month of the diagnosis of disseminated Kaposi’s sarcoma. He died before chemotherapy could be commenced.

**Discussion:**

The lessons that could be learned from this case include the following: Kaposi’s sarcoma is asymptomatic and, since one out of three are HHV-8 positive, patients should have a thorough examination before starting on highly-active antiretroviral therapy. Patients with Kaposi’s sarcoma or even those on treatment should be warned of deterioration in the first 12 weeks of treatment. Pulmonary Kaposi’s sarcoma is fatal and requires timeous management and chemotherapy. Patients with HIV-related Kaposi’s sarcoma and chest signs require sputa to exclude pulmonary tuberculosis. Finally, traditional healers may be used to assist, especially if they are taught to identify HIV-related skin conditions and can refer patients appropriately.

## Abstrait

Rapport de cas sur la propagation rapide du sarcome de Kaposi chez un patient atteint du VIH

**Introduction:**

Le sarcome de Kaposi est la maladie néoplasique associée au VIH la plus courante. Dans la plupart des cas, elle commence sur la peau puis se propage à d’autres organes viscéraux. Nous avons rapporté un cas de sarcome de Kaposi cutané associé au VIH ayant rapidement progressé pour atteindre les organes viscéraux en quelques semaines et ayant entraîné le décès du patient.

**Présentation du cas:**

Un homme de 21 ans qui a récemment entamé une thérapie antirétrovirale a développé un sarcome de Kaposi disséminé avec un épanchement pleural sur le coté droit. Une radiographie des poumons a confirmé l’épanchement qui a été prélevé à des fins de diagnostic. La biopsie a confirmé le sarcome de Kaposi. Le patient a insisté pour pouvoir sortir de l’hôpital afin de consulter un guérisseur traditionnel.

**Prise en charge et résultat:**

Malgré le suivi d’une thérapie antirétrovirale et une prise en charge favorable, l’état du patient s’est rapidement détérioré et il est décédé dans le mois ayant suivi le diagnostic du sarcome de Kaposi disséminé. Il est décédé avant que la chimiothérapie ne puisse être entamée.

**Discussion:**

Les leçons que nous pouvons tirer de ce cas sont notamment les suivantes: Le sarcome de Kaposi est asymptomatique, et étant donné qu’un sur trois est positif au HHV-8, les patients devraient faire l’objet d’un examen minutieux avant de commencer une thérapie antirétrovirale hautement active. Les patients atteints du sarcome de Kaposi ou même ceux sous traitement doivent être tenus informés de la déterioration au cours des 12 premières semaines de traitement. Le sarcome de Kaposi pulmonaire est fatal et exige une prise en charge et une chimiothérapie dans les temps. Les patients atteints du sarcome de Kaposi associé au VIH et de signes dans la poitrine doivent fournir des crachats afin d’exclure la tuberculose pulmonaire. Enfin, les guérisseurs traditionnels peuvent être appelés en renfort afin d’apporter une aide, en particulier si on leur apprend à identifier les maladies de peau associées au VIH et à orienter les patients de manière adéquate.

## Introduction

A young man of 21 years was referred to the level one (Family Medicine) ward of Dr George Mukhari Hospital (DGMH) on 17 May 2010 and died on 7 July 2010. At the time of admission he was fully ambulant and complained of genital ulcers, diarrhoea and oral lesions. However, whilst in the ward he deteriorated rapidly and showed symptoms and signs of disseminated Kaposi’s sarcoma. We will present the case followed by a discussion of Kaposi’s sarcoma in relation to the patient. The focus will be on the management of disseminated Kaposi’s sarcoma.

## Ethical considerations

Ethical approval for the study was obtained from the MEDUNSA research and ethics committee: MREC/M/74/2012:IR. There are no details that will identify the patient concerned. 

## Case presentation

A 21-year old African male patient (Mr NC) presented at the Accident and Emergency Department of DGMH on 17 May 2010. He had mouth sores, skin and genital lesions and diarrhoea for one month and had been coughing for two weeks. 

His medical history revealed that he was HIV positive and had been on highly-active antiretroviral therapy (HAART) since 15 April 2010. He was on retrovir (AZT), lamivudine (3TC) and nevirapine.

On arrival at the Accident and Emergency Department on 17 May 2010, he looked ill and uncomfortable and was markedly pale. His blood pressure (BP) was 114/66 mmHg, pulse rate 112 beats per minute, respiratory rate (RR) 20 breaths per minute and temperature 37.4 °C. His random capillary blood glucose was 5.4 mmol/L. The casualty officer also reported that there were dark skin lesions over the trunk and limbs of the patient, hyperpigmented plaques on the hard palate and the presence of penile and peri-anal warts.

The casualty officer made an assessment of HIV with Kaposi’s sarcoma lesions on the skin and oral cavity and genital warts and then referred the patient to the level one ward of DGMH. 

## Management and outcome 

The plan of management at that stage was to biopsy the skin lesions and refer for a dermatology consultation. The patient was put on oral antibiotics: Zinnat (cefuroxime), Erythromycin and Bactrim (trimethoprim/sulphamethoxazole), with Fluconazole for the oral thrush and a Diclofenac injection for the generalised body pains. The HAART was continued. 

**FIGURE 1:**
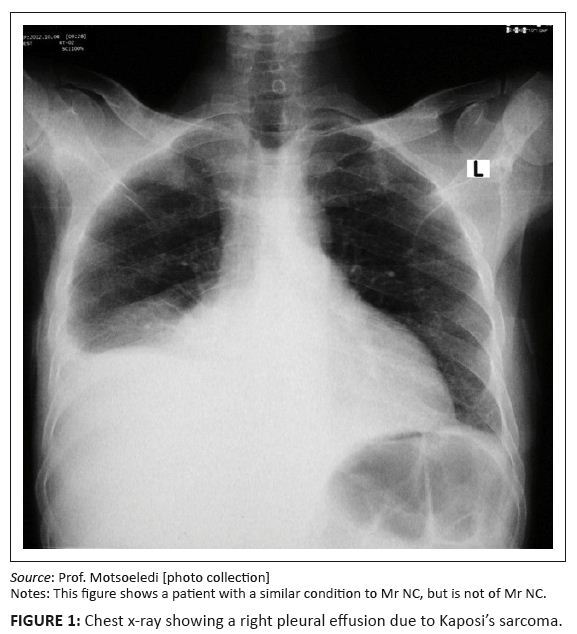
Chest x-ray showing a right pleural effusion due to Kaposi’s sarcoma. *Source:* Prof. Motsoeledi [photo collection] Notes: This figure shows a patient with a similar condition to Mr. NC, but is not of Mr. NC.

On the next day in the ward the patient was reported to be clinically stable. On 19 May 2010 the HIV enzyme-linked immunosorbent assay (ELISA) test was positive and the CD4+ count was 125 cells/cm^3^. Chest x-rays showed right-sided massive pleural effusion (Figure 1 shows typical effusion as seen in cases of Kaposi’s sarcoma). The pleural tap revealed the following results: ADA (adenosine deaminase) 0.9 U/L, total protein 34 g/L and LDH (lactate dehydrogenase) 99 IU/L (S-LDH (serum-LDH) 228 IU/L, transudate ratio <0.4). The haemoglobin was 9.6g/dL, white cell count 5.5 cells/cm^3^, urea 8.8 mmol/L, creatinine 256 µmol/L, total protein 62 g/L, albumin 22 g/L, ALP (alkaline phosphatase) 42 U/L, and gamma-glutamyl transpeptidase (Gamma GT) 48 U/L. The rest of the results (FBC [full blood count], LFTs [liver function tests] and electrolytes) were normal. Biopsy of the skin lesions confirmed Kaposi’s sarcoma. On the same day, ENT (ear, nose and throat) consultation confirmed bilateral serous otitis media. The patient was started on Augmentin (amoxicillin and clavulanate), Exocin (ofloxacin) eardrops and Actifed (antihistamine and nasal decongestion). The pleural fluid was tapped for diagnostic purposes only.

On 24 May 2010, the patient developed anasarca and Furosemide 80 mg orally, daily was started. Sputa were also collected and tested for pulmonary tuberculosis (PTB). On 27 May 2010, PTB treatment was started and a pleural biopsy taken. 

On 29 May 2010, the patient became confused and disorientated. He was aggressive and then bit a nurse in attendance (who was subsequently put on prophylactic ARVs [antiretroviral theory]). The anasarca worsened, with marked sacral oedema. Blood pressure was 90/60 mmHg, pulse 80 beats/min, temperature 37 °C, RR 20 breaths per minute. He was sedated with Ativan (lorazepam). 

On 30 May 2010, he went into respiratory distress with nasal flaring and tachypnoea (RR 38 breaths/min). He was put on oxygen. 

On 2 June 2010, histology of the pleura confirmed Kaposi’s sarcoma. On 3 June, his clinical condition improved. He was ambulant, orientated and eating well. The internists consulted with the patient on 4 June 2010 and reported as follows: AIDS stage 4 with CD4+ count of 125 cells/cm^3^ and disseminated Kaposi’s sarcoma.

Arrangements were made on 4 June 2010 to transfer the patient to an oncology unit. The oncologists reported that the patient had a poor prognosis and chemotherapy was planned. They indicated that patients usually improved on HAART and added Aldactone (spironolactone) and Coversyl (perindopril) to the treatment.

On 4 June 2010, the oncology unit advised that since the diagnosis had already been made, the patient had to be on supportive treatment whilst continuing with the HAART at DGMH.

On 2 July 2010, the patient’s clinical condition improved (ascites, respiratory distress and anasarca settled), and he was well orientated. The oncology unit arranged for chemotherapy for the patient on 9 July 2010. However, the patient and his family wanted a discharge from DGMH so that he could be taken home to KwaZulu Natal. The family wanted to get help from traditional healers in addition to the medical treatment. He reported that he would come back for chemotherapy. The patient’s brother telephoned the Family Medicine Department on 7 July 2010 with the information that his brother had died on that day. He had developed severe respiratory distress and was taken to a primary health care clinic in KwaZulu Natal where he died.

Kaposi’s sarcoma usually progresses slowly and responds to chemotherapy and radiotherapy. Patients with HIV-associated Kaposi’s sarcoma should be started on HAART. Family physicians and other primary health care practitioners need to be able to identify Kaposi’s sarcoma and visceral dissemination needs to be suspected in these immune-compromised patients. Through this case study we explored reasons for this unusual rapid progression of disseminated Kaposi’s sarcoma. 

## Discussion

Kaposi’s sarcoma is the most common neoplastic disease associated with HIV.^1^ It is a multicentric angioproliferative neoplasm, primarily affecting mucocutaneous tissues, but may affect viscera as well.^1^ The oncogenic virus human herpes virus type 8 (HHV-8) is the aetiological agent associated with the development of Kaposi’s scarcoma.^2^ There are four clinical types of Kaposi’s sarcoma: the classic type, African endemic type, HIV-associated type and Kaposi’s sarcoma associated with forms of immunosuppression other than HIV. In South Africa the HIV-associated type is the most common. 

Pathogenesis of Kaposi’s sarcoma in HIV-seropositive subjects involves interaction between HHV-8 and altered cellular signal transduction pathways, increased production of cytokines and growth factors,^1^ thus HHV-8 is a necessary but insufficient cause of Kaposi.^3^ HHV-8 is transmitted sexually and through saliva.^4^ In South Africa, one in three adults is HHV-8 seropositive and the risk of developing Kaposi’s sarcoma is increased more than 20-fold with HIV infection.

**FIGURE 2:**
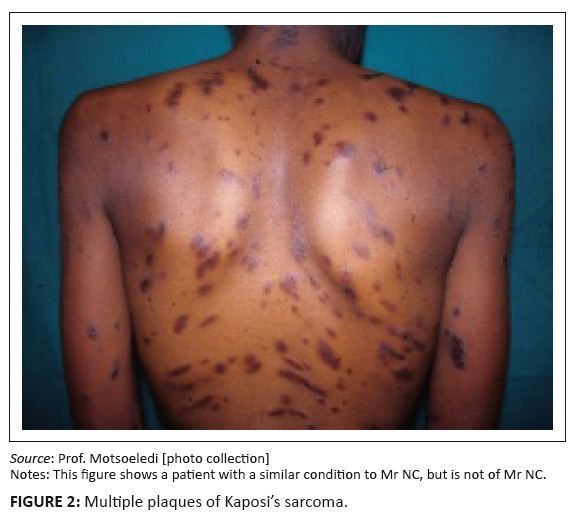
Multiple plaques of Kaposi’s sarcoma. *Source:* Prof. Motsoeledi [photo collection] Notes: This figure shows a patient with a similar condition to Mr. NC, but is not of Mr. NC.

**FIGURE 3:**
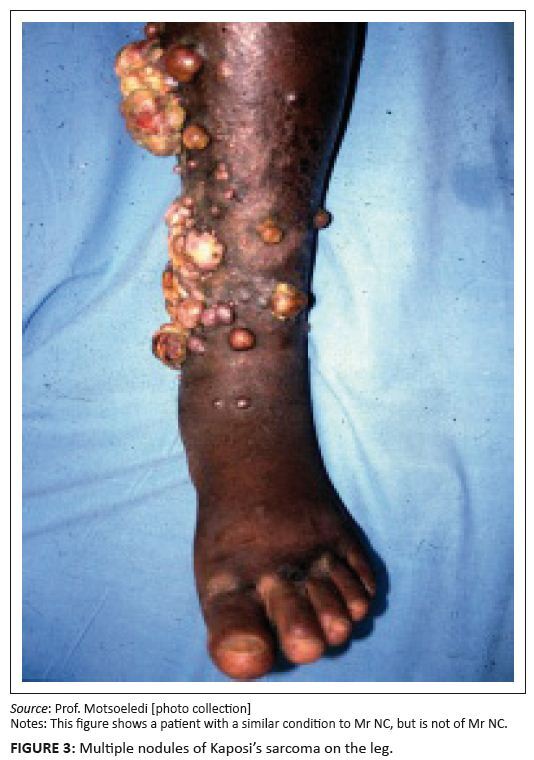
Multiple nodules of Kaposi’s sarcoma on the leg. *Source:* Prof. Motsoeledi [photo collection] Notes: This figure shows a patient with a similar condition to Mr. NC, but is not of Mr. NC.

Clinically, cutaneous Kaposi’s sarcoma presents as discrete erythematous or bluish macules, most commonly on the lower extremities.^5^ The bluish macules may develop into plaques and then nodules. Figures 2 and 3 show multiple plaques and nodules in a patient with Kaposi’s sarcoma. Lymphoedema of the involved areas is common. In the majority of patients, Kaposi’s sarcoma starts on the skin and remains localised to the skin or disseminates to extracutaneous sites. Common extracutaneous sites are the oral cavity, lungs, liver, intestines and lymph nodes.^5^ Other rarer sites include the pancreas, spleen, testes, kidneys, adrenals, urinary bladder and thyroid.^5^

In the lungs, Kaposi’s sarcoma presents with signs of mass lesion and massive pleural effusion. In the liver, it mainly presents with hepatomegaly and ascites. The most common presentation in the intestines is gastrointestinal bleeding. The clinical course of Kaposi’s sarcoma ranges from indolent disease restricted to the skin to rapidly-progressive extensive skin disease with visceral organ involvement leading to organ dysfunction and mortality.^5^ Mr NC developed a massive right-sided pleural effusion whilst he was in the ward. 

In a subset of subjects who are HIV seropositive, Kaposi’s sarcoma may recrudesce early following the introduction of HAART, as the immune system reconstitutes.^6^ In Mr NC’s case, HAART was started on 15 April 2010, but this did not seem to improve his outcome.

Management of HIV-associated Kaposi’s sarcoma is aimed at reduction of intensive skin disease, shrinkage of problematic oral lesions, abatement of pain and alleviation of symptomatic visceral disease. Management involves the use of HAART, radiotherapy and chemotherapy.^1^ HAART is aimed at reducing plasma HIV viral load and restoration of the host immune system and helps in bringing about regression of Kaposi’s sarcoma lesions. Radiotherapy is suitable for treatment of limited skin disease whilst palliative chemotherapy is reserved for extensive skin disease and visceral involvement.^1^

The rapid progression may have been immune reconstitution inflammatory syndrome (IRIS)-related. HIV-infected patients with Kaposi’s sarcoma may develop a fatal reaction after initiating HAART due to IRIS.^7^ IRIS is observed in patients who demonstrate a good virologic and immunologic response to HAART but experience a paradoxical clinical worsening. In IRIS patients, the rapid restoration of functionally-active antigen-specific cells after HAART initially leads to an immunopathologic rather than a protective effect, resulting in worsening of a known condition (paradoxical IRIS) or an atypical presentation of unrecognised opportunistic infections (unmasking IRIS). IRIS has been related to a growing number of infectious, autoimmune and neoplastic manifestations, including tuberculosis, nontuberculous mycobacteria, cryptococcus, herpes viruses, and Kaposi’s sarcoma.^8^

The majority of fatalities are due to pulmonary involvement.^7^ In a study conducted in Mozambique, 11.6% of patients on HAART with Kaposi’s sarcoma developed immune reconstitution inflammatory syndrome-associated with Kaposi’s sarcoma (IRIS-KS).^8^ 

Important take-home messages for the primary care physician from this patient’s case report are that: 

- Kaposi’s sarcoma is asymptomatic and, since one out of three are HHV-8 positive, patients should have a thorough examination before starting HAART.
- In patients with Kaposi’s sarcoma or even those on HAART, they should be warned of deterioration within the first 12 weeks of treatment. 
- Pulmonary Kaposi’s sarcoma is fatal and requires chemotherapy.
- Pulmonary IRIS-KS requires timeous management.
- Patients with HIV-related Kaposi’s sarcoma and chest signs require sputa to exclude PTB.
- Traditional healers can be used to assist, especially if they are taught to identify HIV-related skin conditions and can refer patients appropriately.

## Conclusion

In this case study, we presented an unusually rapid progression of Kaposi’s sarcoma. Within six weeks of the biopsy and confirmation of the diagnosis, the patient had died, despite being on HAART. Patients with Kaposi’s sarcoma have been reported to have an overall five-year relative survival rate of 90%.^9^
